# Submillisecond X-ray photon correlation spectroscopy from a pixel array detector with fast dual gating and no readout dead-time

**DOI:** 10.1107/S1600577516005166

**Published:** 2016-04-19

**Authors:** Qingteng Zhang, Eric M. Dufresne, Pawel Grybos, Piotr Kmon, Piotr Maj, Suresh Narayanan, Grzegorz W. Deptuch, Robert Szczygiel, Alec Sandy

**Affiliations:** a Advanced Photon Source, Argonne National Laboratory, Lemont, IL 60439, USA; b AGH University of Science and Technology, av. Mickiewicza 30, Krakow 30-059, Poland; c Fermi National Accelerator Laboratory, Batavia, IL 60510, USA

**Keywords:** pixel array detector (PAD), high frame rate, photon counting, two counters, X-ray photon correlation spectroscopy (XPCS), small-angle X-ray scattering (SAXS)

## Abstract

Small-angle scattering X-ray photon correlation spectroscopy (XPCS) studies were performed using a novel photon-counting pixel array detector with dual counters for each pixel. Each counter can be read out independently from the other to ensure there is no readout dead-time between the neighboring frames. A maximum frame rate of 11.8 kHz was achieved. Results on test samples show good agreement with simple diffusion. The potential of extending the time resolution of XPCS beyond the limit set by the detector frame rate using dual counters is also discussed.

## Abstract 2

X-ray photon correlation spectroscopy is performed using a dual counter pixel array detector at a frame rate of 11.8 kHz with no readout dead-time.

## 1. Introduction   

The prevalence of high intensity, partially coherent synchrotron X-ray sources worldwide has led to great advances in the development of coherent X-ray scattering techniques, including transmission and Bragg geometry X-ray ptychography (Rodenburg *et al.*, 2007 ▸; Hruszkewycz *et al.*, 2012 ▸), coherent diffraction imaging (Robinson & Harder, 2009 ▸) and X-ray photon correlation spectroscopy (XPCS) (Dierker *et al.*, 1995 ▸; Mochrie *et al.*, 1997 ▸). XPCS measures fluctuations at the mesoscale *via* changes in the coherent scattering patterns from samples. XPCS is derived from the well established dynamical light scattering (DLS) technique, where a laser beam scatters from fluctuations in the dielectric constant of the sample and generates a scattering pattern with sharp angular intensity variations (a so-called ‘speckle’ pattern) due to interference of the scattered radiation. Dynamics in the system are measured from the temporal decorrelation of the speckle patterns. As an extension of DLS into the X-ray regime, XPCS benefits from both the ability of X-ray beams to (weakly) scatter from materials that are opaque to visible light (Sutton *et al.*, 1991 ▸) and to probe length scales as small as 1 Å (Leitner *et al.*, 2009 ▸; Ruta *et al.*, 2014 ▸). Access to a wide dynamic range in delay times is also critically important and has enabled investigations of novel length-scale-dependent dynamics in soft condensed matter (Falus *et al.*, 2006 ▸; Lu *et al.*, 2008 ▸). Studies of dynamics in various systems all benefit from rapid, high fidelity recording of fluctuating speckle patterns and this need will become increasingly important with proposed multibend achromat upgrades of the European Synchrotron Radiation Facility (ESRF) and Advanced Photon Source (APS) set to increase the time-averaged coherent flux by up to two orders of magnitude.

**Figure 1:**
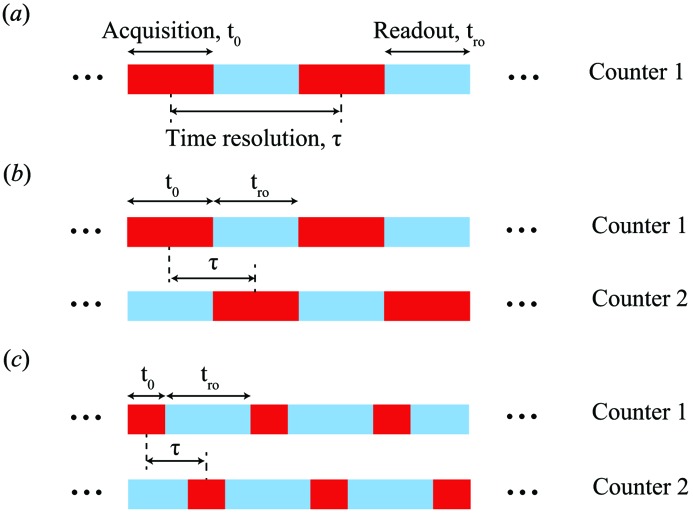
Time infrastructure of (*a*) continuous acquisition with one counter per pixel, (*b*) dual counter acquisition discussed in this study with no readout dead-time between the frames and (*c*) future upgrade of dual counter acquisition where the separation between the frames is smaller than the digitization time associated with each counter.

For multi-speckle XPCS, the time resolution is most typically limited by the detector frame rate, as the frame readout time (the reciprocal of frame rate) is the minimum time separation between the detector frames (Fig. 1 ▸ *a*). Currently, the fastest ‘few’ X-ray-sensitive frame rates are provided by the AGIPD detector (Becker *et al.*, 2011 ▸, 2013 ▸; Schwandt *et al.*, 2013 ▸) and the Keck-PAD (Philipp *et al.*, 2016 ▸). Both of these devices are integrating detectors with the former being developed for the European X-ray Free-Electron Laser (XFEL) and the latter being developed for both synchrotron and FEL applications. The AGIPD is capable of operating at a 4.7 MHz frame rate whereas the Keck-PAD can operate at up to 10 MHz. In both cases, however, only a limited number of frames can be collected before a longer delay for readout and storage which results in too limited a dynamic range for typical XPCS measurements at a synchrotron source. To date, the fastest continuous frame rate tested for multi-speckle XPCS is 22 kHz from the research version of the Eiger detector (Johnson *et al.*, 2012 ▸). Though very fast, the Eiger detector has one counter per pixel and 5 µs of readout dead-time between frames. The dead-time limitation can be removed and also more novel acquisition strategies can be implemented when two (or more) counters are available for each pixel. For instance, both the newly developed Pixirad (Bellazzini *et al.*, 2013 ▸, 2015 ▸) and Lambda (Pennicard *et al.*, 2012 ▸, 2013 ▸) detectors have dual counters assigned to each pixel that can be assigned to provide either ‘color’ vision when different thresholds are applied to each pixel or to provide nearly readout dead-time-free operation when the counters are alternately acquiring or digitizing data (Fig. 1 ▸ *b*). In the readout dead-time-free operation mode, the throughput for the Pixirad is 40 Mpix s^−1^, whereas for the Lambda it is 1.5 Gpix s^−1^. Another novel capability provided by dual counters is, with sufficiently fast gating and sensor response dead-time, the ability to resolve two closely spaced signals in time, *i.e.* recording the sample scattering immediately before and after a laser impulse. This capability is provided by a prototype detector developed by Voxtel (Ross *et al.*, 2016 ▸). Very recently, the applicability of this detector for short delay time XPCS has also been explored (Dufresne *et al.*, 2016 ▸).

Here we present XPCS results obtained using the new Ultra Fast X-ray Camera 32k (UFXC32k) which is a single-photon-counting PAD with dual counters that can operate at a frame rate as high as 11.8 kHz (0.4 Gpix s^−1^). The dual counters can be read out alternately so there is essentially no readout dead-time between neighboring frames. Though the total pixel throughput is not yet as high as that for the Lambda, this detector provides the shortest delay times of any dual counter detector and with anticipated near-term upgrades (see below) will provide a total pixel throughput comparable with the Lambda and a minimum delay time approximately a factor of two better than was achieved with the research version of the Eiger. To test the applicability of this detector for fast time-resolved coherent scattering, XPCS measurements were performed on latex nanoparticles suspended in glycerol/water and the results were in good agreement with simple diffusion. Our work demonstrates the capacity of the UFXC32k to perform XPCS experiments with very high frame rates and no readout dead-time.

## 2. Instrumentation   

**Figure 2:**
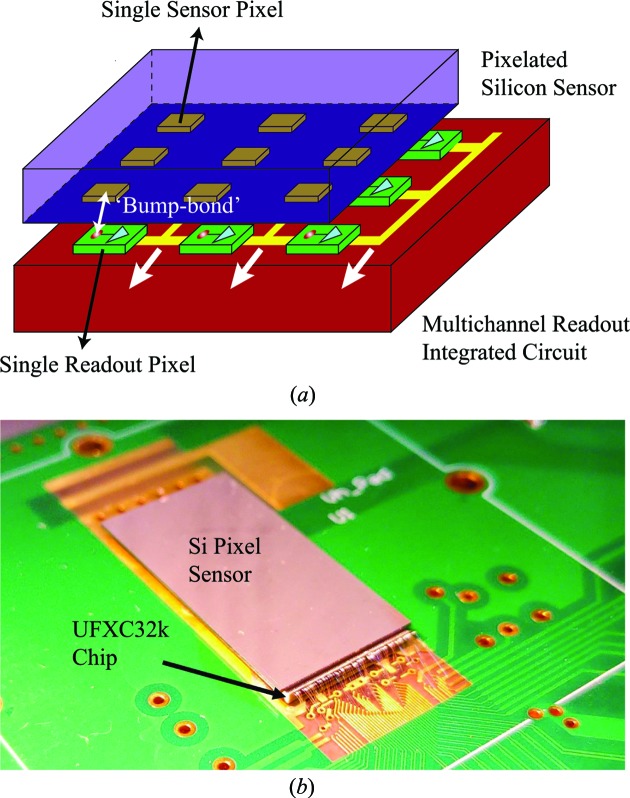
(*a*) Illustration of the pixelated architecture of the Si PN diode sensor and the readout chip of UFXC32k. (*b*) Photo of UFXC32k.

**Figure 3:**
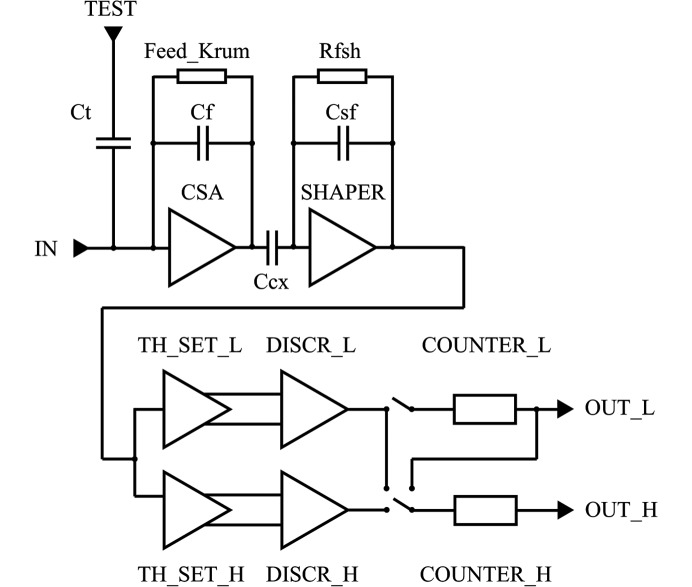
Schematic of the readout circuit of a single pixel.

**Figure 4:**
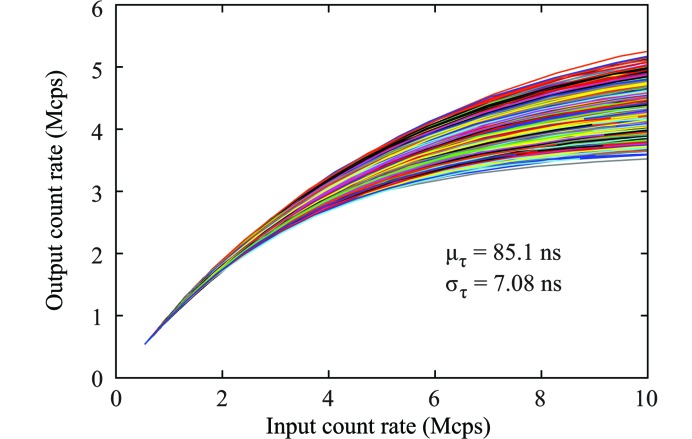
Dead-time measurement for about 1200 pixels of UFXC32k.

The detector has two parts: a reverse-biased pixelated silicon PN diode as the sensor (128 × 256 square-shaped pixels, pixel size of 75 µm and thickness of 320 µm) and a readout integrated circuit with built-in pixel architecture (see Fig. 2 ▸ *a*). Each pixel on the PN diode is bump-bonded to its own dedicated electronics. X-ray photons arrive at the depleted region of the PN diode and generate electron–hole pairs. Fig. 3 ▸ is the schematic of the readout circuit of a single pixel. The current signal from the collected electron–hole pairs under the reverse bias voltage is integrated into a charge-sensitive preamplifier (CSA). The preamplifier outputs a voltage pulse with its amplitude proportional to the total charge generated in the pixel. The voltage pulse is then fed to the main amplifier (SHAPER), which reshapes the pulse based on the specifications of noise filtering and timing. If the amplitude of the reshaped pulse is above the preset threshold (TH_SET_L and TH_SET_H), a photon count is recorded in the counter. With respect to charge sharing, like other PADs, during typical operation the threshold is set to half the mean pulse amplitude of a pixel-isolated event. In this way, all photon hits are recorded except for those near the vertex of four pixels (the slope = 1 on the left-hand side of Fig. 4 ▸ indicates a very small number of lost events). With the use of trim digital-to-analog converters in each pixel, the effective threshold spread at the discriminator input is only 8.5 e^−^ r.m.s. For the nominal gain of about 50.3 µV e^−^ the measured gain spread is only 1.9% (standard deviation/mean). As a result, no flat-field correction is required, which is not typically the case for PAD detectors (Maj *et al.*, 2014 ▸; Grybos *et al.*, 2016 ▸). The measured noise for the nominal settings is 123 e^−^ r.m.s.

The dead-time of a detector pixel is defined as the time window after a registered photon event within which all arrival photon events will be ignored (Müller, 1991 ▸). The pixel dead-time of UFXC32k is calibrated using a high-power copper-target rotating-anode X-ray generator. Fig. 4 ▸ shows the relation between the count rate measured by the detector and the rate of the X-ray photons impinging on the detector. The ratio between the measured and the incoming X-ray flux is nearly 1 for all pixels up to 10^6^ counts per pixel per second, which is 10^3^ times stronger than even the strongest scattering intensity in this study. The dead-time is evaluated using the paralyzable dead-time model (Müller, 1991 ▸; Walko *et al.*, 2010 ▸):
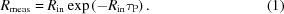
Here, 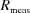 is the measured count rate, 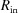 is the averaged incoming X-ray pulse rate and 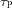 is the pixel dead-time. The 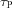 of the UFXC32k detector has a mean of 85.1 ns and a standard deviation of 7.08 ns. So far the shortest pixel dead-time reported from a PAD is 67 ns measured on the integrated circuit of the PILATUS3 with instant retrigger capability and a pixel size of 172 µm × 172 µm (Loeliger *et al.*, 2012 ▸). In the UFXC32k detector the dead-time can be as low as 85 ns and, given that the pixel area is 5.2 times smaller (75 µm × 75 µm), the count rate per detector unit area is significantly higher than that of the PILATUS3.

During the data readout phase, the counters in each column of the pixel array form a shift register. The data from the register is loaded bit by bit into the peripheral fast 128-bit registers and shifted out of the UFXC32k chip *via* eight LVDS parallel lines. Each pixel has two discriminators (DISCR_L and DISCR_H with thresholds) and two 14-bit ripple counters (COUNTER_L and COUNTER_H). The counters in a pixel can operate in two different modes. The first one is the dual-counter mode, in which each counter is connected to a different discriminator. The level of thresholds (TH_SET_L and TH_SET_H) can be set to different values to allow for energy discrimination or the same value to perform fast acquisition with no readout dead-time. The second one is the long counter mode, in which the two counters are linked serially to form one 28-bit long counter for measurements that require very large dynamic range. The readout dead-time of a single frame 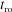 can be expressed in the following equation:
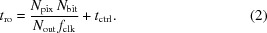
Here, 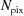 = 128 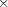 256 = 32768 is the total number of pixels, 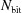 is the bit depth of the pixel (can be switched between 2, 4, 8 or 14 bits), 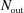 = 8 is the number of LVDS parallel lines, 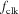 = 100 MHz is the clock frequency of the circuit and 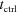 = 2.56 µs is the additional time required for starting the readout sequence at 100 MHz clock frequency. During the experiment, 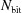 is set to 2 (the dynamic range of the detector is from zero to 3) to achieve the minimum 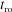 of 84.48 µs and an ultrafast frame rate of 11.8 kHz. The probability of a pixel receiving four photons in the region of interest with the strongest scattering intensity is measured to be 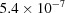 in this study, which is equivalent to 0.017 pixels per frame. We therefore do not expect significant truncation of the scattering intensities. The acquisition time *t*_0_ for the detector frame is set to be equal to 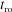 to maximize the counting statistics. As long as *t*_0_ is greater than 2 µs for both counters, the switching time between counters is shorter than the bunch separation time at the APS (153 ns), leading to essentially no readout dead-time between the neighboring frames (Fig. 1 ▸ *b*). A more detailed list of detector parameters is given by Grybos *et al.* (2016 ▸).

## 3. Experiment and discussion   

Small-angle X-ray scattering (SAXS) measurements were performed at the experimental station 8-ID-I of the APS. Two Undulator As were set to produce 7.4 keV X-rays. A water-cooled double-crystal Ge(111) monochromator selected a monochromatic beam with a bandpass of about 0.03% and a 20 µm × 150 µm (H×V) slit was used to select the coherent part of the beam, which had a flux of approximately 2 × 10^10^ photons s^−1^. The beam was then vertically focused at the sample with a full width at half-maximum (FWHM) of about 3 µm using 10 one-dimensional parabolic compound refractive X-ray lenses made of beryllium with a radius of curvature of 0.2 mm (RX Optics). Guard slits between the lens and the sample were used to remove background scattering from both the lenses and the blades on the main collimating slit. A tungsten beamstop immediately upstream from the detector (4 m downstream of the sample) blocked the direct beam.

**Figure 5:**
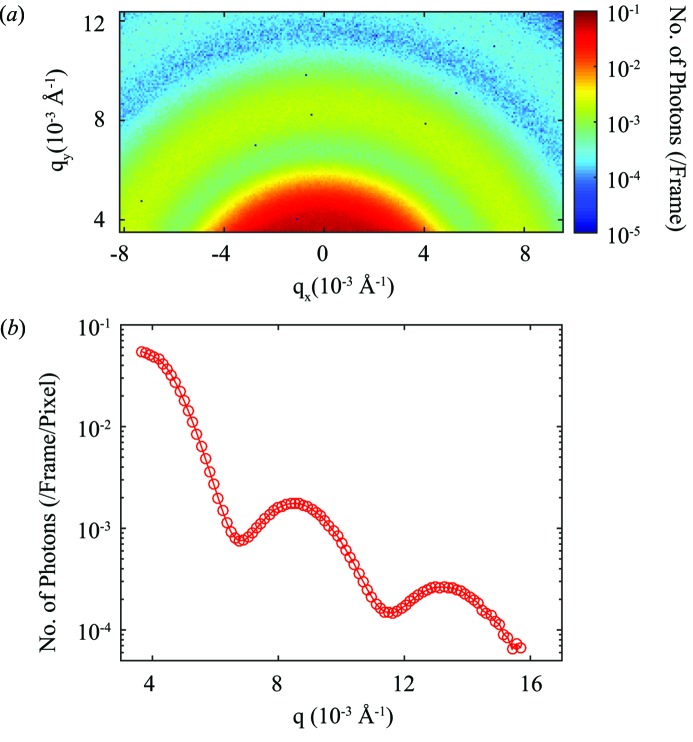
(*a*) Time-averaged scattering from the latex nanoparticle suspension. The scattering intensity is indicated by the logarithmic color bar. (*b*) Azimuthal average of Fig. 5 ▸(*a*).

The sample is 4% (volume fraction) 67 nm-radius latex nanoparticles dispersed in glycerol (Lurio *et al.*, 2000 ▸). The solvent may contain traces of water. The sample was sealed in 1 mm-diameter glass capillaries, integrated into the vacuum of the experiment setup and measured at room temperature. During each acquisition sequence, a series of 100000 frames of coherent scattering patterns are collected from the sample in about 8.5 s. The pixels were converted into coordinates in reciprocal space based on the sample-to-detector distance, the photon energy and the transverse position of the detector pixels relative to the direct beam. A total of nine acquisition sequences were performed to improve the correlation function statistics. Fig. 5 ▸(*a*) shows the time-averaged two-dimensional small-angle X-ray scattering pattern from the sample. The number of dead pixels is only 10 out of a total of 32768 pixels. Fig. 5 ▸(*b*) is the azimuthal average of Fig. 5 ▸(*a*) plotted as a function of *q*. The average radius of the particles calculated from the spacing between the fringes is 66.7 nm, which is very close to the expected value of 67 nm.

**Figure 6:**
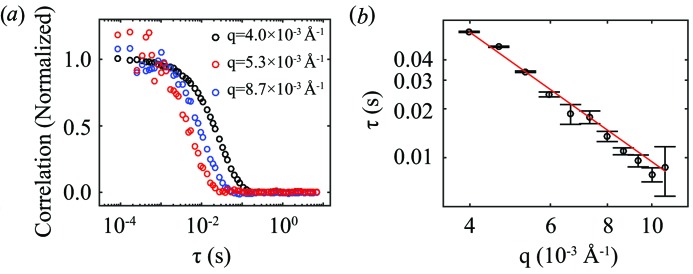
(*a*) Dynamics of latex nanoparticles indicated by *g*_2_(τ) at different *q*. (*b*) Decorrelation time τ(*q*) *versus* *q*. The red line shows the inverse-square decay of the correlation time.

The dynamics of the latex nanoparticles are determined from the correlation coefficient *g*_2_(τ) between speckle patterns separated by a time τ, which is defined as:
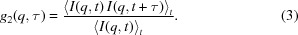
Here, *I*(*q*,*t*) is the photon count of a pixel at wavevector *q* in the frame collected at time *t* and *g*_2_ is evaluated within the multi-τ framework (Cipelletti & Weitz, 1999 ▸) for improved efficiency of the calculation. The time average 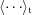 goes over all pairs of frames with a temporal separation of τ. The statistics of *g*_2_ are improved by first azimuthally averaging over pixels with similar *q* to calculate *g*_2_ for each data set and then averaging the *g*_2_ values from the nine data sets. The correlation time τ_0_(*q*) can be extracted from fitting the following equation to the measured correlation decays:
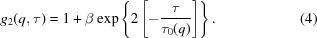
Here, β is the instrumental beamline contrast that depends on the pixel size, speckle size and beamline factors, and is measured to be about 10% for this experiment. It is clear from equation (4) that *g*_2_ decays to the baseline of 1 at long delay times. Fig. 6 ▸(*a*) shows *g*_2_(τ) calculated using equation (3) at a few different values of *q*. The baseline (at 1) has been subtracted and the resulting correlation decays have been normalized by the short-time contrast. It can be seen from Fig. 6 ▸(*a*) that the sample decorrelates faster at smaller length scales (larger *q* values).

For particles undergoing Brownian motion, the correlation time and the wavevector *q* at which it is measured satisfy the inverse-square relation:
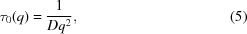
where *D* is the diffusion coefficient. The *q* dependence in Fig. 6 ▸(*b*) is fitted using an inverse-square function. The good agreement between experimental results and the prediction for simple diffusion demonstrates the capacity of the UFXC32k detector to capture fast dynamics with high fidelity. Further comparison between the calculated and the measured diffusion constant is not possible (nor the point of this paper) owing to the ambiguity of the solvent viscosity from traces of water mixed in the glycerol.

## 4. Conclusion   

The UFXC32k is a photon-counting dual-counter PAD with a very fast frame rate of 11.8 kHz. The dual-counter setup can be used for energy discrimination, expansion of the dynamic range or frame acquisition with zero readout dead-time (Fig. 1 ▸ *b*). In addition, both the length and position of the gating signal for dual counters are fully adjustable, meaning that the minimum temporal separation between acquisitions is not restricted by the digitization time of one of the counters (Fig. 1 ▸ *c*). In this case, XPCS studied using PADs with time resolution nearing 1 µs is possible given enough coherent flux (Dufresne *et al.*, 2016 ▸). Future upgrades will include: (i) increasing the frame rate to 47.2 kHz by using a 400 MHz clock signal; (ii) reducing the data transfer time and increasing the largest number of frames allowed in each acquisition through sparsification (saving only pixels with non-zero readouts). With anticipated increases in coherent flux from synchrotron X-ray sources becoming a global trend, *i.e.* the recent start-ups of Petra-III and NSLS-II and the expected future upgrades of the ESRF and APS, development and implementation of fast frame rate, dual counter detectors such as the UFXC32k will surely help expand the capacity of XPCS and open doors to considerable scientific opportunities.
